# Implant treatment modalities of non-syndromic congenitally missing premolars: a retrospective case series of 74 specialist care patients over an 11-year-cohort

**DOI:** 10.2340/aos.v84.44657

**Published:** 2025-09-04

**Authors:** Sarwat Jabeen Hassan, Olli-Pekka Lappalainen, Marleena Ojala-Alasuutari, Virpi Harila, Ritva Näpänkangas

**Affiliations:** a Research Unit of Population Health, Faculty of Medicine, University of Oulu, Oulu, Finland; b Department of Oral and Maxillofacial diseases, Faculty of Medicine, University of Helsinki and Helsinki University Hospital, Helsinki, Finland; c Medical Research Center, Oulu University Hospital and University of Oulu, Oulu, Finland

**Keywords:** Hypodontia, oligodontia, tooth aplasia, tooth agenesis, congenitally missing teeth, dental implants, premolars

## Abstract

**Objective:**

The study aims to evaluate the course of implant treatment for congenitally missing lower second premolars in patients referred to publicly funded specialist care over an 11-year period.

**Material and methods:**

This retrospective, register-based cohort study selected patient data on dental implant treatment in the lower second premolar region from 2009 to 2019 (*n* = 146). After applying exclusion criteria, the final sample included 74 patients. Data on retained deciduous teeth, orthodontic treatment prior to implant placement, and implantation procedures were gathered from patient files. Results were presented using descriptive statistics, t-test, and Chi-square analyses (*p* < 0.05).

**Results:**

The most common orthodontic treatment was space optimization before dental implant placement. Most retained deciduous teeth were extracted between ages 21 and 29, with a median of 6 months before implant placement. Bone grafting was performed at 23% of implant sites. The mean implant diameter was 4.0 mm, and the mean implant length was 9.6 mm. Good primary stability was achieved in all cases.

**Conclusion:**

Individually planned orthodontic and surgical treatments are essential in managing congenitally missing lower premolars, particularly when implant placement is indicated. Early diagnosis and collaboration between dental specialists are key elements in the treatment. A multidisciplinary approach ensures optimal space for implant, bone preservation, and optimal implant alignment.

## Introduction

Congenitally missing teeth, or hypodontia, is a malformation where one or more teeth do not develop as expected. It can affect primary or permanent teeth and is considered one of the most common polymorphisms in humans [1, 2]. Hypodontia can be classified as mild to moderate when one to five teeth are missing, severe hypodontia or oligodontia when six or more teeth are missing, and anodontia when all permanent teeth are absent [1]. Excluding the third molars, mandibular premolars are the most commonly congenitally missing permanent teeth, accounting for around 60–72% of the total number of missing teeth [2]. Additionally, studies have confirmed that females tend to have more missing teeth than males [3, 4]. Bilateral congenitally missing teeth are also more common than unilateral missing teeth [5, 6].

Dental implants have proven to be favorable therapeutic options for treating partially or fully edentulous spaces, including in patients with congenitally missing teeth [7, 8]. Other treatment options include solely orthodontic treatments, such as orthodontic space closing or orthognathic surgeries to address skeletal problems associated with hypodontia [9]. Preservation of deciduous teeth, autotransplantation, conventional prostheses, fixed partial dentures, or removable prostheses are also some remedies used in treatment [10].

When treating mild to moderate hypodontia, dental implants have shown adequate success in improving masticatory ability, esthetics, and dental rehabilitation with long-term survival [11, 12]. Thus, dental implants have become the treatment of choice for restoring all forms of hypodontia at some point during the treatment course. Implants can be placed only in patients who show clinical signs of dental and skeletal growth cessation [13]. However, there are limitations in placing dental implants in hypodontia patients due to dentoalveolar features. The dentoalveolar process is typically narrow in hypodontia patients because no permanent teeth develop at the site, or due to retained deciduous teeth with root resorption, which does not maintain the alveolar bone. There may also be a deficient horizontal volume of soft tissue. In such cases, surgical bone augmentation or bone dilatation is required to facilitate the implant and avoid fenestration of the implant surface or dehiscence [13]. Maintaining the vertical length of bone is important to accommodate the optimal length of the dental implant. Alveolar bone volume can be compromised due to the failure of tooth development or the loss of deciduous teeth followed by residual ridge resorption [13]. In cases of missing mandibular premolars, the inferior alveolar neurovascular bundle is important and at risk of impingement if the optimal implant length is pursued [13]. Another consideration for implant placement is the mesio-distal space. Radiographic assessments should be made prior to implant placement to analyze the space between the roots of adjacent teeth.

Ankylosis and infraocclusion are more common reasons than caries for the loss of retained deciduous second molars with no permanent successor [7]. The frequency of infraocclusion of retained mandibular deciduous molars is 10 times higher compared to those in the maxilla [14]. Additionally, ankylosis commonly occurs in primary mandibular second molar sites [15]. Root resorption is often observed in retained deciduous teeth, although it is of less clinical significance [16, 17]. There is an abrupt and accelerated rate of alveolar bone resorption both vertically and horizontally within 6 months of deciduous tooth extraction. Planned atraumatic extraction and immediate provisionalization can provide pleasing aesthetic results while preserving the tooth gingival contour, especially when dental implants are intended as a treatment [16, 18].

Hypodontia or oligodontia cannot be managed solely by conservative prosthetic means, as they may not provide completely satisfactory results. Depending on the severity of the hypodontia, a multidisciplinary approach in specialist care is often needed, involving pediatric dentistry, periodontology, restorative dentistry, orthodontics, oral surgery, and prosthodontics [11, 19, 20]. In Finland, the Ministry of Social Affairs and Health has established nationwide standards for non-emergency dental care, which include guidelines for treating congenitally missing teeth. According to these standards, dental care is provided free of charge through local primary health care services for individuals under 18 years old. For all age groups, dental care in government-funded specialist care is heavily subsidized.

The aim of the study was to evaluate treatment patterns before implant treatment in patients with congenitally missing lower premolars. For this purpose, the types of orthodontic treatment modalities administered before implant placement as well as the timing and reasons for deciduous tooth extractions were evaluated in patients with congenitally missing lower second premolars.

## Material and methods

This study is retrospective, and register based. Permission to conduct the study was granted by the Northern Ostrobothnia Hospital District (project number 130/2019). The sample consisted of patients from the Northern Ostrobothnia Hospital District who were referred to the Department of Oral and Maxillofacial Surgery at Oulu University Hospital due to congenitally missing teeth and were treated between January 1, 2009 and October 27, 2019.

### Inclusion and exclusion criteria

**Figure 1:**
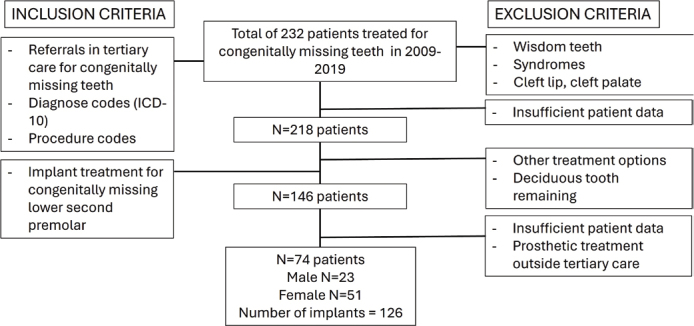
Flow chart of the data for the selected patients with the inclusion and exclusion criteria.

The patients were selected by searching the data of Oulu University Hospital for the ICD10 (International Classification of Diseases-10) diagnosis code K00.00 partial anodontia (hypodontia, oligodontia), and with the procedure codes EBA00 (extraction of tooth), EBA05 (difficult extraction of tooth), EBA10 (surgical tooth extraction), EBA12 (surgical tooth extraction, difficult), EBB10 (implantation), and EBB20 (auto-transplantation) [21]. Exclusion criteria adopted were missing third molars, syndromes, cleft lip, and cleft palate. The search yielded 232 patients who were having congenitally missing teeth [21] (Figure 1).

Out of 232 patients, data for 14 patients did not provide accurate information on congenitally missing teeth, reducing the total number of patients to 218. From these, patients who had received dental implants in the lower second premolar region were selected and included in the present study (*n* = 146). Further exclusion criteria were applied to patient data that did not provide sufficient information or had missing data on the variables of interest for this study, or where the prosthetic crowns were made in primary or secondary care outside the hospital, although the implants had been placed in specialist care.

The total number of patients in this study was 74, of which 51 (69%) were female and 23 (31%) were male. At the time of referral, the patients’ ages ranged from 13 to 57 years (mean 29.5 years), and at the time of implantation, from 18 to 57 years (mean 31.1 years). Patients had total of 126 congenitally missing lower second premolars, which have been treated with dental implants.

Patients had 1 to 19 congenitally missing teeth (Table 1). In most cases, patients had 2 (23%) or 4 (14.9%) congenitally missing teeth, and 63.6% of the patients suffered from hypodontia (1–5 teeth missing). Bilaterally missing teeth were more common (78%) than unilaterally missing teeth (22%).

**Table 1:** Number of congenitally missing teeth (third molars excluded) in 74 patients referred to specialist care for treatment in 2009–2019.

Number of congenitally missing teeth	Female (*n* = 51)		Male (*n* = 23)		Total (*n* = 74)	
		
	*n*	%	*N*	%	*n*	%
1	2	3.9	3	13.0	5	6.8
2	8	15.7	9	39.1	17	23.0
3	6	11.8	0	0	6	8.1
4	8	15.7	3	13.0	11	14.9
5	7	13.7	1	4.3	8	10.8
6	6	11.8	0	0	6	8.1
7	4	7.8	3	13.0	7	9.5
8	4	7.8	1	4.3	5	6.8
9	1	2.0	0	0	1	1.4
10	2	3.9	2	8.7	4	5.4
11	0	0	1	4.3	1	1.4
13	1	2.0	0	0	1	1.4
19	2	3.9	0	0	2	2.7

Orthodontic treatment options prior to implant placement during hospital treatment were: (1) no treatment; (2) occlusal rehabilitation in cases of orthognathic surgeries for open and deep bites and sagittal malocclusion problems; (3) space optimization for implants; and (4) space maintenance for implants. In addition, the orthodontic treatment performed before specialist care was registered.

Retained deciduous teeth were registered as being extracted either (1) before referral, (2) during hospital treatment before implant placement, or (3) during the implant placement operation. The date of extraction and the reason for extraction of deciduous teeth (root resorption, ankylosis and infra-occlusion, caries, or fracture) were also recorded.

All the implants were medical-grade commercially pure titanium or titanium alloys. The length and width of the implant were recorded. Implant cases were evaluated based on hard and soft tissue grafts, graded as follows: (1) implant placement with no graft; (2) implant placement requiring hard tissue grafting procedures (intraoral and extraoral harvesting grafts); and (3) implant placement requiring soft tissue grafting procedures. The date of implant placement was also registered.

## Methods

The orthodontic treatment modalities were compared with age of the patients and the phase of the extraction of deciduous teeth. The elapsed time between extraction of deciduous tooth (lower second deciduous molar) and implant placement was counted and the time period was compared with orthodontic treatment modalities as well as implant width, length and grafting procedures needed.

### Statistics

The results are presented as prevalences in tables and figures. Differences between variables were tested using *t*-test and Chi-square test. IBM SPSS Statistics version 27 was used for analysis. Any *p* value less than 0.05 was considered statistically significant.

## Results

Prior to implant placement in patients with congenitally missing lower second premolars, the most common orthodontic treatments were space optimization for implants (45.9%) and occlusal rehabilitation (28.4%) (Table 2). No orthodontic treatment was needed for 16/74 patients (21.6%). Orthodontic treatment was most often performed for patients aged 20–29 years (39.7%) or 30–39 years (26.0%). No significant differences existed between the orthodontic treatment modalities and age of the patients (*p* = 0.252).

**Table 2:** Orthodontic treatment performed in different age groups among 74 patients referred to specialist care for treatment of congenitally missing lower second premolars in 2009–2019.

Age group	No treatment	Occlusal rehabilitation	Space optimization for implants	Space maintenance	Total
				
	*n* (%)	*n* (%)	*n* (%)	*n* (%)	*n* (%)
< 20 years (*n* = 9)	0 (0)	3 (30.0)	7 (70.0)	0 (0)	10 (13.5)
20–29 years (*n* = 29)	7 (24.1)	7 (24.1)	13 (44.8)	2 (6.9)	29 (39.2)
30–39 years (*n* = 19)	7 (36.8)	5 (26.3)	6 (31.6)	1 (5.3)	19 (25.7)
40–49 years (*n* = 11)	1 (9.1)	4 (36.4)	6 (54.5)	0 (0)	11 (14.9)
> 50 years (*n* = 5)	1 (20.0)	2 (40.0)	2 (40.0)	0 (0)	5 (6.7)
Total	16 (21.6)	21 (28.4)	34 (45.9)	3 (4.1)	74 (100)

**Figure 2:**
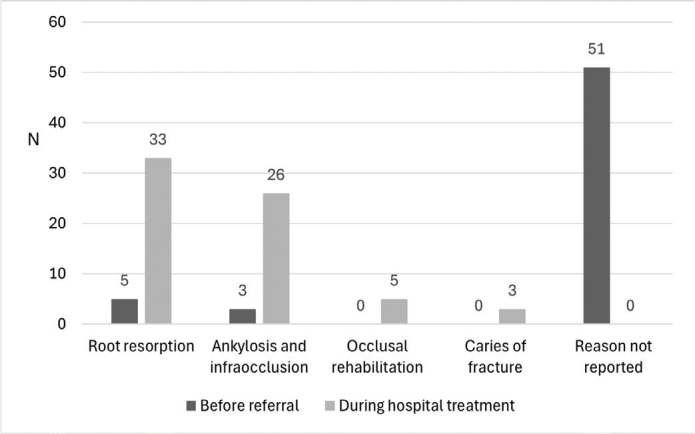
The indication for extraction of deciduous teeth (*n* = 126) in 74 patients referred to specialist care for treatment of congenitally missing lower second premolars over the period 2009–2019. The number of root resorption includes the deciduous teeth extracted before and during the implant placement during hospital treatment.

Deciduous teeth were extracted quite evenly either before referral (47.6%) or during the hospital treatment period before implant placement (50.8%) (Table 3). The most common reasons for the extraction of deciduous teeth were root resorption or ankylosis and infraocclusion (Figure 2).

**Table 3:** The extraction status of deciduous teeth (*n* = 126) before implant placement in region of congenitally missing second premolar in different age groups among 74 patients referred to specialist care for treatment in 2009–2019.

Age group (number of deciduous teeth)	Extracted before referral	Extracted before implant placement	Extracted during implant placement
		
	*n* (%)	*n* (%)	*n* (%)
< 20 years (*n* = 17)	5 (29.4)	12 (70.6)	0 (0)
21–29 years (*n* = 50)	24 (48.0)	26 (52.0)	0 (0)
30–39 years (*n* = 33)	17 (51.5)	16 (48.5)	0 (0)
40–49 years (*n* = 19)	10 (52.6)	9 (47.4)	0 (0)
> 50 years (*n* = 7)	4 (57.1)	1 (14.3)	2 (28.6)
Total (*n* = 126)	60 (47.6)	64 (50.8)	2 (1.6)

**Figure 3:**
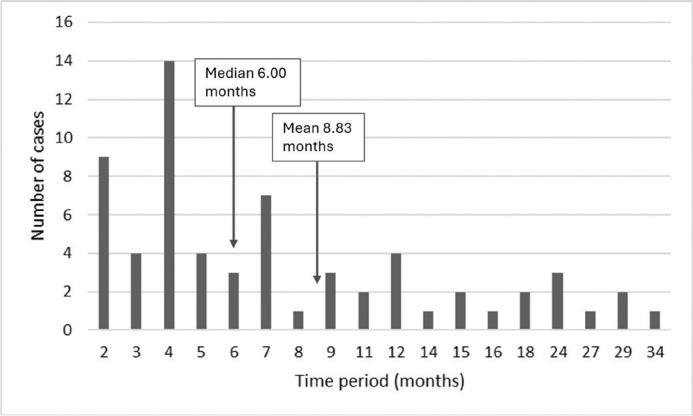
Time period between extraction of deciduous tooth and implantation among 74 patients referred to specialist care for treatment of hypodontia or oligodontia over the period 2009–2019. Number of deciduous teeth with known time of extraction *n* = 64, no information *n* = 62.

The median time from the extraction of a deciduous tooth to implant placement was 6.00 months (mean 8.83 months, range 2–34 months) (Figure 3). The time period (6 months or less/more than 6 months) was associated significantly (*p* = 0.010) with orthodontic treatment modalities. When the time period was 6 months or less, the most common treatments were space optimization for implants or no orthodontic treatment (Table 4). When the time period was more than 6 months, the most usual orthodontic treatments were space optimization or occlusal rehabilitation.

**Table 4:** The orthodontic treatment performed in relation to the extraction status of deciduous teeth before implant placement in region of congenitally missing second premolar among 74 patients referred to specialist care for treatment in 2009–2019. Number of deciduous teeth with known time of extraction *n* = 64, no information *n* = 62.

Orthodontic treatment	Time period between extraction of deciduous tooth and implant placement	

	≤ 6 months	> 6 months
	
	*n* (%)	*n* (%)
No treatment (*n* = 15)	13 (86.7)	2 (13.3)
Occlusal rehabilitation (*n* = 12)	5 (41.7)	7 (58.3)
Space optimization for implants (*n* = 33)	16 (48.5)	17 (51.5)
Space maintenance (*n* = 4)	0 (0.0)	4 (100)
Total	34 (53.1)	30 (46.9)

The mean implant diameter was 4.0 mm (range 3.3–4.8 mm) and the mean implant length was 9.6 mm (range 8.0–13.0 mm). Bone grafting procedures were indicated at 23% (*n* = 29/126) of the implant sites for congenitally missing lower second premolars (Table 5). In these cases, autogenous grafts and bovine bone xenografts with membranes were most often used. Primary stability of the implants was good in all cases. No soft tissue grafts were indicated. The implant width, implant length, or bone grafting procedures needed for implants were not associated with the timing between extraction of lower second deciduous molar and implant placement.

**Table 5:** Bone grafts indicated and width of the implants in treating congenitally missing lower second premolars (*n* = 126).

Width of implant (mm)	No bone graft	Intraoral harvested bone graft	Extraoral harvested bone graft
		
	*n* (%)	*n* (%)	*n* (%)
3.3	8 (8.2)	7 (29.1)	2 (40)
3.4	3 (3.1)	0	1 (20)
3.6	6 (6.2)	1 (4.2)	0
3.8	15 (15.5)	5 (20,8)	1 (20)
4.1	38 (39.2)	10 (41.7)	1 (20)
4.2	14 (14.4)	1 (4.2)	0
4.5	4 (4.1)	0	0
4.8	9 (9.3)	0	0
Total	97 (100)	24 (100)	5 (100)

## Discussion

This study included 74 patients with congenitally missing lower premolars who received dental implants as a treatment for congenitally missing teeth in specialist care. Bilateral missing teeth were present in 78% of the cases, which corresponds to other studies [5, 6]. Patients with congenitally missing teeth were not only treated with dental implants but for most patients, orthodontic intervention was indicated, and in some cases, bone augmentation procedures were performed before implant placement. Oulu University Hospital is a tertiary level center of excellence for practicing senior and specializing dentists. Individual treatment choices and plans are discussed by employing a multidisciplinary approach and including orthodontists, prosthodontists, periodontists, pediatric dentists, and oral and maxillofacial surgeons for patients in need, as mentioned in other studies [11, 19, 20]

A total of 78.4% of the patients underwent orthodontic treatment before implant placement. The time period between extraction of deciduous tooth and implant placement was associated significantly with orthodontic treatment modalities. When the time period exceeded 6 months, more patients required space optimization or occlusal rehabilitation compared to those with a time period 6 months or less. Orthodontic treatment also included occlusal rehabilitation, addressing deep bite, overjet and overbite corrections, adjusting crowding in the lower frontal area, and surgical correction of hypoplastic maxilla and mandible through orthognathic surgeries.

The prognosis of retained deciduous teeth without permanent successors is somewhat unpredictable, and the right time to extract the retained tooth due to implant treatment would be important to know. In this study, the retained deciduous teeth persisted in patients ranging from 20 to 69 years of age. In most cases, the retained deciduous teeth were kept in place until they were extracted just before implant placement (median 6 months), although in half of the patients, deciduous teeth were extracted before referral to Oulu University Hospital and the definitive diagnosis for extraction was not known. Root resorption, ankylosis, and infra-occlusion were reported as dominant features associated with the retained deciduous teeth. Related to implant treatment, root resorption is typically of less clinical significance, as root resorption is a physiological phenomenon in deciduous dentition although pathological in permanent dentition [22]. More important than root resorption are ankylosis and infraocclusion. In many patient cases, by the age of 10–11, a primary molar may already exhibit severe infraocclusion, root resorption, or caries, necessitating the tooth’s extraction. In some situations, severe crowding of the dental arch can be diagnosed early, allowing for the extraction decision to be made during the second transitional phase [23]. The decision to extract a primary molar sometimes has to be made at an older age, during or after a growth spurt, when infraocclusion is very severe and a vertical bone defect has developed [15, 24]. However, primary molars with mild infraocclusion, diagnosed early, should be monitored regularly every 6 months. If infraocclusion becomes severe, the clinician should consider extracting the primary molar, ideally before the growth spurt. This can prevent significant vertical bone defects. A substantial deficiency in the alveolar bone later poses challenges, for example, in replacing the permanent tooth with an implant [15, 23, 24,].

In case of infra-occlusion, adjacent teeth erupt normally as the alveolar process develops and grows naturally [25, 26] In that case, clinical findings include tipping of adjacent teeth, overeruption of opposing teeth, apical positioning of the gingival margins, lateral open bites, and incomplete development of the alveolar process in the region [14]. All these conditions are unfavorable for the placement of dental implants, and orthodontic treatment is indicated before implant treatment [9].

The implant length, diameter or grafting procedures needed were not associated with the timing between extraction of lower second deciduous molar and implant placement. The mean length of 9.6 mm, and the mean diameter of 4.0 mm for implants was achieved, which can be considered acceptable. Horizontal bone corrections were performed in 23% of the cases using autogenous and bovine bone grafts before implant placement, which is a lower proportion than in upper jaw incisal region implant sites [27]. In addition, extraoral harvested grafts were used in three patients with severe hypodontia (in the region of five implants). The study emphasized that clinicians use the 2.9–3.3 mm narrow diameter dental implants in the rehabilitation of congenitally missing lateral incisors, which is also related to favorable aesthetics and high patient satisfaction with the implant treatment [28, 29].

Vertical bone corrections may also be needed to protect the neurovascular bundle in the mandible, which is of great anatomical significance while restoring congenitally missing premolars [13]. Another challenge for implant placement is the mesio-distal space of the osseous alveolar ridge [13]. Horizontal graft procedures or bone dilatation procedures are performed to enable the placement of an optimally sized implant [13]. Implants lack a periodontal ligament and are prone to stress breakage [30]. For this reason, the importance of the implant diameter cannot be overlooked. Soft tissue grafts may also be needed in some cases to avoid dehiscence in the alveolar process. Furthermore, tapered implants can be considered in cases of insufficient augmentation results [13]. To add more keratinized tissue or width to the attached gingiva, an apically repositioned flap or vestibuloplasty procedures combined with autogenous soft tissue grafts can be used. More soft tissue volume gain can also be achieved by using subepithelial connective tissue grafts rather than free gingival grafts [31].

The present study evaluated the implant treatment of congenitally missing second premolars. When more teeth are missing, oral rehabilitation of congenitally missing teeth becomes more challenging [32, 33]. The challenges are related to the lack of width and height of the alveolar bone [10]. The absence of teeth at the site prevents normal horizontal and vertical development of dentoalveolar tissues [13]. Moreover, if an implant is placed in vertically deficient bone, it results in poor aesthetics and unfavorable biomechanics due to a high crown-to-implant ratio [13]. Radiographic evaluation is crucial for dental implant planning to avoid damaging vital structures [34]. Cone beam computed tomography (CBCT) aids in precise virtual implant placement by visualizing the height, width, volume, and morphology of the alveolar bone, as well as the proximity to adjacent teeth and vital structures [35, 36]. 3D implant planning software allows for detailed planning of implant location, angle, depth, and diameter [37].

This study focuses on the evaluation and treatment of patients with congenitally missing lower second premolars in Northern Ostrobothnia, Finland, from 2009 to 2019. The strengths of the study include its long duration (11 years) and extensive data on a wide age range of patients referred to publicly funded specialist dental care. The data are based on comprehensive data collection and include detailed information on various aspects of patient treatment before implant treatment. In addition, a multidisciplinary approach was possible to evaluate. The limitation of the study is that it was conducted at only one center, and the study is based on a specific population from the Northern Ostrobothnia Hospital District and patients treated at Oulu University Hospital. This may limit the generalizability of the findings to other populations or regions with different healthcare systems or demographic characteristics. Further, the study is retrospective, which may introduce biases related to the accuracy and completeness of the recorded data. The data are based on existing records, which were collected with the current aim resulting in some missing or incomplete information. In further study it would be interesting to evaluate patients’ satisfaction and outcomes of implant treatment in patients with congenitally missing teeth, because complications may relate to less satisfaction with the treatment and poorer oral function [38]. In addition, further research could also compare the implant treatment of congenitally missing maxillary premolar sites versus mandibular premolar sites.

Based on the findings of the current study, it is advisable to retain the primary molars for as long as possible, ideally until the patient reaches an appropriate age for implant placement, if the treatment plan involves replacing the missing premolar with an implant. The longevity of the primary molar can be extended with restorative treatments, thereby supporting adequate bone preservation and optimal implant positioning.

Early identification of congenitally missing premolars is essential for effective long-term planning. For pediatric dentists and general practitioners managing adolescent patients with congenitally missing primary second molars, interdisciplinary consultation with an orthodontist is often necessary to establish a comprehensive occlusal treatment plan. For instance, in cases of severe crowding, extraction of the primary second molar may be a strategic option to alleviate space issues. Furthermore, before proceeding with early extraction of the primary second molar, it is recommended that an orthodontist be consulted to determine an appropriate follow-up treatment plan (e.g. space maintenance, space closure, or alternative options).

In conclusion, individually planned orthodontic and surgical treatments are essential in managing congenitally missing lower premolars, particularly when implant placement is indicated. Early diagnosis and collaboration between dental specialists are key elements in the treatment. A multidisciplinary approach ensures optimal space for implant, bone preservation, and optimal implant alignment.

## References

[1] Nunn JH, Carter NE, Gillgrass TJ, Hobson RS, Jepson NJ, Meechan JG, et al. The interdisciplinary management of hypodontia: background and role of paediatric dentistry. Br Dent J. 2003;194(5):245–51. 10.1038/sj.bdj.4809925. 12658298

[2] Polder BJ, Van’t Hof MA, Van der Linden FPGM, Kuijpers-Jagtman AM. A meta-analysis of the prevalence of dental agenesis of permanent teeth. Community Dent Oral Epidemiol. 2004;32(3):217–26. 10.1111/j.1600-0528.2004.00158.x 15151692

[3] Carter K, Worthington S. Morphologic and demographic predictors of third molar agenesis: a systematic review and meta-analysis. J Dent Res. 2015;94(7):886–894. 10.1177/0022034515581644 25883107

[4] Rakhshan V, Rakhshan A. Systematic review and meta-analysis of congenitally missing permanent dentition: sex dimorphism, occurrence patterns, associated factors and biasing factors. Int Orthod. 2016;14(3):273–94. 10.1016/j.ortho.2016.07.016 27522615

[5] Heuberer S, Ulm C, Zechner W, Laky B, Watzak G. Patterns of congenitally missing teeth of non-syndromic and syndromic patients treated at a single-center over the past thirty years. Arch Oral Biol. 2019;1(98):140–7. 10.1016/j.archoralbio.2018.11.018 30496934

[6] Bergström K. An orthopantomographic study of hypodontia, supernumeraries and other anomalies in school children between the ages of 8–9 years. An epidemiological study. Swedish Dent J. 1977;1(4):145–157. 270232

[7] Laverty DP, Fairbrother K, Addison O. The current evidence on retaining or prosthodontically replacing retained deciduous teeth in the adult hypodontia patient: a systematic review. Eur J Prosthodon Restorat Dent. 2018;26(1): 2–15. 10.1922/EJPRD_01721Laverty14 29461745

[8] Allen PF, Lee S, Brady P. Clinical and subjective evaluation of implants in patients with hypodontia: a two‐year observation study. Clin Oral Implants Res. 2017;28(10):1258–62. 10.1111/clr.12951 27502579

[9] Borzabadi-Farahani A. Orthodontic considerations in restorative management of hypodontia patients with endosseous implants. J Oral Implantol. 2012;38:779–91. 10.1563/AAID-JOI-D-11-00022 21728818

[10] Terheyden H, Wüsthoff F. Occlusal rehabilitation in patients with congenitally missing teeth-dental implants, conventional prosthetics, tooth autotransplants, and preservation of deciduous teeth – a systematic review. Int J Implant Dent. 2015;1:30. 10.1186/s40729-015-0025-z 27747652 PMC5005685

[11] Burns B, Grieg V, Bissell V, Savarrio L. A review of implant provision for hypodontia patients within a Scottish referral centre. Br Dent J. 2017;21(223):96–9. 10.1038/sj.bdj.2017.623 28729571

[12] Filius MAP, Vissink A, Cune MS, Raghoebar GM, Visser A. Long-term implant performance and patients’ satisfaction in oligodontia. J Dent. 2018;71:18–24. 10.1016/j.jdent.2018.01.007 29360491

[13] Jepson NJ, Nohl FS, Carter NE, Gillgrass TJ, Meechan JG, Hobson RS, et al. The interdisciplinary management of hypodontia: restorative dentistry. Br Dent J. 2003;22(194):299–304. 10.1038/sj.bdj.4809940 12682653

[14] Arhakis A, Boutiou E. Etiology, diagnosis, consequences and treatment of infraoccluded primary molars. Open Dent J. 2016;10:714–9. 10.2174/1874210601610010714 28217186 PMC5299554

[15] Hua L, Thomas M, Bhatia S, Bowkett A, Merrett S. To extract or not to extract? Management of infraoccluded second primary molars without successors. Br Dent J. 2019;227(2):93–8. 10.1038/s41415-019-0207-9 31350491

[16] Hvaring CL, Øgaard B, Stenvik A, Birkeland K. The prognosis of retained primary molars without successors: infraocclusion, root resorption and restorations in 111 patients. Eur J Orthod. 2014;36:26–30. 10.1093/ejo/cjs105 23314329

[17] Dos Santos CC, Melo DL, da Silva PP, Normando D. What is the survival rate of deciduous molars in cases with agenesis of premolar successors? A systematic review. Angle Orthodon. 2022;92(1):110–7. 10.2319/123020-1039.1 PMC8691466 34329385

[18] Tavarez RR, Dos Reis WL, Rocha AT, Firoozmand LM, Bandéca MC, Tonetto MR, et al. Atraumatic extraction and immediate implant installation: the importance of maintaining the contour gingival tissues. J Int Oral Health. 2013;5:113–18. 24453455 PMC3895728

[19] Breeze J, Dover MS, Williams RW. Contemporary surgical management of hypodontia. Br J Oral Maxillofac Surg. 2017;55:454–60. 10.1016/j.bjoms.2017.03.013 28410841

[20] Pace-Balzan A, Chatzipantelis A, J Dunn K, Charan G, P Ashley M. Restorative dentistry clinical decision-making for hypodontia: complex cases. Br Dent J. 2023;235:489–95. 10.1038/s41415-023-6324-5 37828181 PMC10570138

[21] Ojala-Alasuutari M, Hassan SJ, Näpänkangas R, Ylikontiola L, Lähdesmäki R. Distribution of congenitally missing teeth and treatment options for the lower second premolars in patients referred to special care. Acta Odontol Scand. 2022;80:382–8. 10.1080/00016357.2021.2021282 34962856

[22] Patel S, Saberi N, Pimental T, Teng PH. Present status and future directions: Root resorption. Int Endodont J. 2022;55(Suppl 4):892–921. 10.1111/iej.13715 PMC9790676 35229320

[23] Bjerklin K. Orthodontic management of agenesis of mandibular second premolars. APOS Trends Orthod. 2019;9(4):206–10. 10.25259/APOS_122_2019

[24] Sabri R. Management of over-retained mandibular deciduous second molars with and without permanent successors. World J Orthod. 2008;9(3):209–20. 18834004

[25] Aranha AM, Duque C, Silva JY, Carrara CF, Costa B, Gomide MR. Tooth ankylosis in deciduous teeth of children with cleft lip and/or palate. Brazilian Oral Res. 2004;18:329–32. 10.1590/S1806-83242004000400010 16089265

[26] Chong JA, Mah EC. Orthodontic alignment of ankylosed teeth with aid of surgical luxation: case series. J Orthodon. 2024;51(3):283–91. 10.1177/14653125231185362 37401621

[27] Roccuzzo A, Imber JC, Jensen SS. Need for lateral bone augmentation at two narrow-diameter implants: a prospective, controlled, clinical study. Clin Oral Implants Res. 2021;32(4):511–20. 10.1111/clr.13721 33548077

[28] Roccuzzo A, Imber JC, Lempert J, Hosseini M, Jensen SS. Narrow diameter implants to replace congenital missing maxillary lateral incisors: a 1‐year prospective, controlled, clinical study. Clin Oral Implants Res. 2022;33(8):844–57. 10.1111/clr.13966 35763401 PMC9544295

[29] Roccuzzo A, Imber JC, Lempert J, Jensen SS. Clinical, radiographic, and aesthetic outcomes at two narrow‐diameter implants to replace congenital missing maxillary lateral incisors: a 3‐year prospective, clinical study. Clin Implant Dent Relat Res. 2024;26(4):777–86. 10.1111/cid.13339 38863078

[30] Esposito M, Thomsen P, Ericson LE, Sennerby L, Lekholm U. Histopathologic observations on late oral implant failures. Clin Implant Dent Relat Res. 2000;2:18–32. 10.1111/j.1708-8208.2000.tb00103.x 11359271

[31] Thoma DS, Naenni N, Figuero E, Hämmerle CH, Schwarz F, Jung RE, et al. Effects of soft tissue augmentation procedures on peri‐implant health or disease: a systematic review and meta‐analysis. Clin Oral Implants Res. 2018;29:32–49. 10.1111/clr.13114 29498129

[32] Créton M, Cune M, Verhoeven W, Muradin M, Wismeijer D, Meijer G. Implant treatment in patients with severe hypodontia: a retrospective evaluation. J Oral Maxillofac Surg. 2010;68:530–8. 10.1016/j.joms.2009.09.012 20171472

[33] Alam MK, Purmal K, Low A, Pohchi A. Interdisciplinary case of multiple congenitally missing permanent teeth. Bangladesh J Med Sci. 2017;16(3):467–73. 10.3329/bjms.v16i3.32878

[34] Salian SS, Subhadarsanee CP, Patil RT, Dhadse PV. Radiographic evaluation in implant patients: a review. Cureus. 2024;16:e54783. 10.7759/cureus.54783 38529466 PMC10961673

[35] Worthington P, Rubenstein J, Hatcher DC. The role of cone-beam computed tomography in the planning and placement of implants. J Am Dent Assoc. 2010;141:19S–24S. 10.14219/jada.archive.2010.0358 20884936

[36] Jacobs R, Salmon B, Codari M, Hassan B, Bornstein MM. Cone beam computed tomography in implant dentistry: recommendations for clinical use. BMC Oral Health. 2018;18(1):88. 10.1186/s12903-018-0523-5 29764458 PMC5952365

[37] Schubert O, Schweiger J, Stimmelmayr M, Nold E, Güth JF. Digital implant planning and guided implant surgery–workflow and reliability. Br Dent J. 2019;226(2):101–8. 10.1038/sj.bdj.2019.44 30679852

[38] Mauland EK, Bull VH, Melbye EL, Verket A. Patient-reported outcomes following dental implant rehabilitation according to reason for missing teeth: a survey from a Norwegian population 8 years following treatment. J Clin Periodontol. 2024;51:135–44. 10.1111/jcpe.13895 37915235

